# Taxonomic, Spatial and Temporal Patterns of Bleaching in Anemones Inhabited by Anemonefishes

**DOI:** 10.1371/journal.pone.0070966

**Published:** 2013-08-08

**Authors:** Jean-Paul A. Hobbs, Ashley J. Frisch, Benjamin M. Ford, Michele Thums, Pablo Saenz-Agudelo, Kathryn A. Furby, Michael L. Berumen

**Affiliations:** 1 The Oceans Institute and School of Plant Biology, The University of Western Australia, Perth, Australia; 2 Australian Institute of Marine Science, The Oceans Institute, Perth, Australia; 3 Australian Research Council Centre of Excellence for Coral Reef Studies, James Cook University, Townsville, Australia; 4 School of Environmental Systems Engineering and The Oceans Institute, The University of Western Australia, Perth, Australia; 5 Red Sea Research Center, King Abdullah University of Science and Technology, Thuwal, Kingdom of Saudi Arabia; 6 Marine Biology Research Division, Scripps Institution of Oceanography, La Jolla, California, United States of America; 7 Biology Department, Woods Hole Oceanographic Institution, Woods Hole, Massachusetts, United States of America; Leibniz Center for Tropical Marine Ecology, Germany

## Abstract

**Background:**

Rising sea temperatures are causing significant destruction to coral reef ecosystems due to coral mortality from thermally-induced bleaching (loss of symbiotic algae and/or their photosynthetic pigments). Although bleaching has been intensively studied in corals, little is known about the causes and consequences of bleaching in other tropical symbiotic organisms.

**Methodology/Principal Findings:**

This study used underwater visual surveys to investigate bleaching in the 10 species of anemones that host anemonefishes. Bleaching was confirmed in seven anemone species (with anecdotal reports of bleaching in the other three species) at 10 of 19 survey locations spanning the Indo-Pacific and Red Sea, indicating that anemone bleaching is taxonomically and geographically widespread. In total, bleaching was observed in 490 of the 13,896 surveyed anemones (3.5%); however, this percentage was much higher (19–100%) during five major bleaching events that were associated with periods of elevated water temperatures and coral bleaching. There was considerable spatial variation in anemone bleaching during most of these events, suggesting that certain sites and deeper waters might act as refuges. Susceptibility to bleaching varied between species, and in some species, bleaching caused reductions in size and abundance.

**Conclusions/Significance:**

Anemones are long-lived with low natural mortality, which makes them particularly vulnerable to predicted increases in severity and frequency of bleaching events. Population viability will be severely compromised if anemones and their symbionts cannot acclimate or adapt to rising sea temperatures. Anemone bleaching also has negative effects to other species, particularly those that have an obligate relationship with anemones. These effects include reductions in abundance and reproductive output of anemonefishes. Therefore, the future of these iconic and commercially valuable coral reef fishes is inextricably linked to the ability of host anemones to cope with rising sea temperatures associated with climate change.

## Introduction

Increasing sea temperatures associated with global warming are having a profound effect on coral reef ecosystems, largely due to coral bleaching [1]. Bleaching can lead to partial or total mortality of corals and can reduce future reproduction and compromise immunity [2]–[4]. Mass bleaching events occur when sea temperatures on a reef remain elevated for prolonged periods of time, and can result in up to 99% coral mortality [5]. High irradiance from solar radiation can also contribute to bleaching in thermally-stressed corals [6]–[7]. To predict how rising sea temperature will affect the community composition and spatial distribution of coral reefs in the future, much research effort has focused on determining spatial, temporal and taxonomic patterns of coral bleaching. This research has revealed that bleaching severity can be species-specific [8], [9] and spatially variable, with some habitats acting as refuges [10]–[13]. Bleaching is usually greatest in shallow waters where sea temperatures and irradiance are highest [12], [14], [15]. In addition to the direct effects on corals, bleaching can also have significant impact to other organisms (e.g. fish) that rely on corals for food and/or shelter [16], [17].

Coral bleaching, so named because affected corals appear bright white, is due to the loss of symbiotic algae and/or symbiont pigments, and usually occurs during elevated sea temperatures and often in association with high solar irradiance [6]. These symbiotic algae (zooxanthellae - *Symbiodinium*) are present in a wide range of taxonomic groups including hard (scleractinian) and soft corals, zoanthids, hydrozoans, molluscs, foraminifera, jellyfish and anemones [18]. Many of these organisms have important ecological functions, such as filtering particulates from the water column, cementing reef substrate or providing habitat for other species. Despite this importance, the effects of bleaching on reef-associated organisms other than scleractinian corals are seldom studied. Elevated sea temperatures are forecast to lead to frequent large-scale bleaching events in scleractinian corals [19], and non-sceleractinian benthic taxa may be equally affected [20]. However, we have little understanding of how bleaching will affect non-scleractinian organisms and what the effects will be to other species and the ecosystem in general.

Several anemone species on coral reefs contain zooxanthellae and are also symbiotic with a range of fish, crab and shrimp species [21], [22]. For some species, such as the iconic anemonefishes, the symbiosis is obligate. Currently, there are 30 known species of anemonefishes, which associate with 10 species of anemones (hereafter referred to as host anemones) [22]–[24]. Host anemones and anemonefishes are of significant commercial importance because they are popular attractions in the coral reef tourism industry and are key fisheries targets in the marine aquarium trade [25], [26]. Host anemones are found on coral reefs throughout suitable parts of the tropical and sub-tropical Indo-Pacific, with distributions of some species extending into the Red Sea. Laboratory experiments have demonstrated that at least one species of host anemone will bleach when water temperatures and irradiance exceed a specific threshold [27]; however, the causes and consequences of bleaching in host anemones remain largely unknown in the natural environment [28], [29].

To enable accurate predictions of how increasing sea temperatures will impact host anemones, we conducted field-based studies of anemone bleaching events to determine the taxonomic, spatial, and temporal patterns of bleaching. This type of study is important to identify whether some species and habitats are more susceptible than others and thus require enhanced conservation and management. Furthermore, monitoring host anemones following bleaching events is necessary to determine changes in size and abundance, and hence assess population viability. We aimed to address these issues by answering the following specific questions:

1. Is anemone bleaching associated with elevated water temperatures?
2. Which species of host anemones bleach?
3. Are there differences between species of host anemones in the proportion of individuals that bleach (i.e. bleaching susceptibility)?
4. Does bleaching occur throughout the geographic ranges of host anemones?
5. Does anemone bleaching vary among sites and depths within a region? That is, are there bleaching refuges at a fine habitat scale?
6. Is there a reduction in the size or number of anemones at a location following bleaching?

## Methods

### Study Species and Sites

To broaden the spatial and temporal context of the study we compiled datasets across multiple locations, years and bleaching events. The inclusion of benthic datasets that were assembled for a variety of purposes meant that a range of underwater visual census approaches were used to count the number of bleached and unbleached host anemones (Table 1). Underwater visual censuses were conducted between 2005 and 2012 across 276 sites at 19 locations in the Pacific Ocean, Indian Ocean, and Red Sea (Table 1). Survey dates were haphazard and were not targeted to periods of elevated water temperature. The survey locations spanned the geographic ranges of all the host anemones [21]. At most locations, surveys were conducted at multiple depths. Censuses focused on all 10 anemone species known to be inhabited by anemonefishes: *Cryptodendrum adhaesivum*, *Entacmaea quadricolor*, *Heteractis aurora, H. crispa*, *H. magnifica, H. malu, Macrodactyla doreensis, Stichodactyla gigantea*, *S*. *haddoni*, and *S. mertensii* [21].

**Table 1:** Survey details for the nineteen study locations.

Location and coordinates	Region	Survey date	Survey methods
Marshall Islands (7.131°N, 171.183°E)	Pacific Ocean	July, 2009	10 sites, 2 depths (3 and 10 m), 3 replicate belt transects (250×5 m) per depth. Sites = haphazardly chosen, outer reef and lagoon. Observer = J-P Hobbs.
		June, 2010	14 sites, 2 depths (3 and 10 m), 3 replicate belt transects (250×5 m) per depth. Sites = haphazardly chosen, outer reef and lagoon. Observer = J-P Hobbs.
Norfolk Island (29.041°S, 167.951°E)	Pacific Ocean	March, 2012	8 sites, 2 depths (5 and 15 m), and 2 sites with 1 depth (2 m). 3 replicate belt transects (50×5 m) per depth. Additional 4 sites with a timed swim (≈480×5 m) at each site at 2 m depth. Sites = haphazardly chosen, outer reef and lagoon. Observer = J-P Hobbs.
Kosrae, Micronesia (5.299°N, 162.973°E)	Pacific Ocean	June, 2010	Timed swims (≈50×5 m) at 5 m depth at each of 4 sites. Sites = haphazardly chosen, outer reef. Observer = J-P Hobbs.
		February, 2012	15 sites at 10 m depth, and another 7 sites at 4 m depth. 3 replicate belt transects (50×5 m) per site. Sites = haphazardly chosen, outer reef. Observer = J-P Hobbs.
Lord Howe Island (31.531°S, 159.067°E)	Pacific Ocean	March, 2009	13 sites at 2 depths (5 and 15 m), 1 site at 5 m depth, and another 9 sites at 2 m depth. 3 replicate belt transects (50×5 m) per depth. Sites = haphazardly chosen, outer reef and lagoon. Observer = J-P Hobbs.
		November, 2011	9 sites with a timed swim (≈520×5 m) surveying depths 15 to 25 m. Sites = haphazardly chosen, outer reef and lagoon. Also, point counts on 9 selected lagoonal patch reefs (≈10 m^2^, 2 m depth) that had high anemone densities. Observer = J-P Hobbs.
Middleton Reef (29.472°S, 159.119°E)	Pacific Ocean	March, 2011	8 sites at 2 depths (3 and 9 m) and another site at 3 m depth. 3 replicate belt transects (50×5 m) per depth. Additional survey on one reef (25×40 m) at depths of 2–10 m. Sites = haphazardly chosen, outer reef and lagoon. Observer = J-P Hobbs.
Elizabeth Reef (29.942°S, 159.058°E)	Pacific Ocean	March, 2011	7 sites at 2 depths (3 and 9 m), with 3 replicate belt transects (50×5 m) per depth. Sites = haphazardly chosen, outer reef and lagoon. Observer = J-P Hobbs.
Pohnpei, Micronesia(6.854°S, 158.289°E)	Pacific Ocean	July, 2009	Timed swim (≈50×5 m) at 5 m depth at 1 site and 2 timed swims at 5 m depth at another site. Sites = haphazardly chosen, outer reef and lagoon. Observer = J-P Hobbs.
North Solitary Island(29.929°S, 153.391°E)	Pacific Ocean	August, 2011	Timed swim (≈50×5 m) at 10–15 m depth at each of 2 sites. Sites = exposed rocky islands, chosen for high anemone density. Observer = J-P Hobbs.
Mooloolaba, Eastern Australia (26.678°S, 153.118°E)	Pacific Ocean	June, 2011	Timed swim (≈840×5 m) at 13–24 m depth at each of 5 sites. Timed swim (≈460×5 m) at 13–21 m depth at another site. Sites = haphazardly chosen, exposed rocky reefs. Observer = J-P Hobbs.
Keppel Islands, Great Barrier Reef (23.178°S, 150.948°E)	Pacific Ocean	October, 2007	13 sites at 3 m depth, 10 sites at 7 m depth, 6 sites at 2 depths (3 and 7 m), 6 sites at 3 depths (3, 7 and 15 m) (total of 35 sites). 1 GPS tracked swim (≈600×5 m) per depth. Sites = haphazardly chosen, exposed and protected fringing reefs on continental islands [40]. Also, point counts at another 5 areas (≈6 m^2^) that were selected due to high densities of anemones. Observers = A. Frisch, J-P Hobbs.
		December, 2011	6 sites at 3 m depth, 2 sites at 7 m depth, 1 site at 3 and 7 m depths, 1 site at 7 and 15 m depths, and 1 site at 3 depths (3, 7 and 15 m) (total of 11 sites). 1 GPS tracked swim (≈600×5 m) per depth. Sites = haphazardly chosen, exposed and protected fringing reefs on continental islands [40]. Also, point counts at 17 other areas (≈6 m^2^) that were selected due to high anemone densities. Observer = A. Frisch
Bootless Bay, Papua New Guinea (9.52°S, 147.28°E)	Pacific Ocean	January to April, 2009	6 sites, 1 survey area (7500–12500 m^2^) per site covering depths of 2 to 20 m. Sites = haphazardly chosen, sandy sheltered areas [38]. Observer = P. Saenz-Agudelo.
Guam (13.446°N, 144.775°E)	Pacific Ocean	July, 2010	Timed swim (≈50×5 m) at 1 depth (5 m) at each of 5 sites. Timed swim (≈420×10 m) at 5 m depth at another site. Sites = haphazardly chosen, outer reef and lagoon. Observer = J-P Hobbs.
Ashmore Reef, Western Australia (12.254°S, 123.050°E)	Indian Ocean	April, 2009	Timed swim (≈300×5 m) at 2 m depth at each of 3 sites. Sites = haphazardly chosen, exposed and protected fringing reefs on continental islands. Observer = J-P Hobbs.
Christmas Island (10.50°S, 105.67°E)	Indian Ocean	May, 2005	5 sites at 2 depths (5 and 20 m). 9 replicate belt transects (50×5 m) at each depth at 3 of the sites and 3 replicates per depth at the other 2 sites. Sites = haphazardly chosen outer reef. Observer = J-P Hobbs.
		May, 2010	7 sites, 2 depths (5 and 20 m), timed swims (≈50×5 m). The number of timed swims per depth ranged from 1 to 7. Sites = haphazardly chosen, outer reef. Observer = J-P Hobbs.
Cocos (Keeling) Islands (12.20°S, 96.90°E)	Indian Ocean	March, 2008	17 sites, 2 depths (5 and 20 m), 3 replicate belt transects (50×5 m) per depth. Sites = haphazardly chosen, outer reef. Observer = J-P Hobbs.
		September, 2008	Point count survey on a patch reef (5×2 m) at 8 m depth that was selected due to a high density of anemones. Observer = J-P Hobbs.
Farasan Banks (17.47–20.55°N, 39.09–41.44°E)	Red Sea	October, 2009	Timed swim (≈200×5 m) at depths from 5 to 20 m at each of 13 sites. Sites = haphazardly chosen, offshore, mid-shelf and inshore reefs. Observers = K. Furby, M. Berumen
Farasan Banks (19.29°N, 40.01°E)	Red Sea	June, 2010	Timed swim (≈200×5 m) at depths from 5 to 20 m at each of 5 sites. Sites = haphazardly chosen, mid-shelf reefs. Observers = K. Furby, M. Berumen
Thuwal-Yanbu (21.41–20.55°N, 38.46–39.03°E)	Red Sea	January, 2010	16 sites, 2 depths (5 and 15 m), 3 replicate belt transects (50×5 m) per depth. Sites = haphazardly chosen, offshore, mid-shelf and inshore reefs. Observers = K. Furby, M. Berumen
Thuwal (22.03–26°N, 38.46–39.03°E)	Red Sea	September, 2010	8 sites, 3 depths (5,10 and 15 m), 3 replicate line intercept transects (length = 10 m) per depth. Sites = haphazardly chosen, offshore, mid-shelf and inshore reefs [43]. Observers = K. Furby, M. Berumen

**Figure 1:**
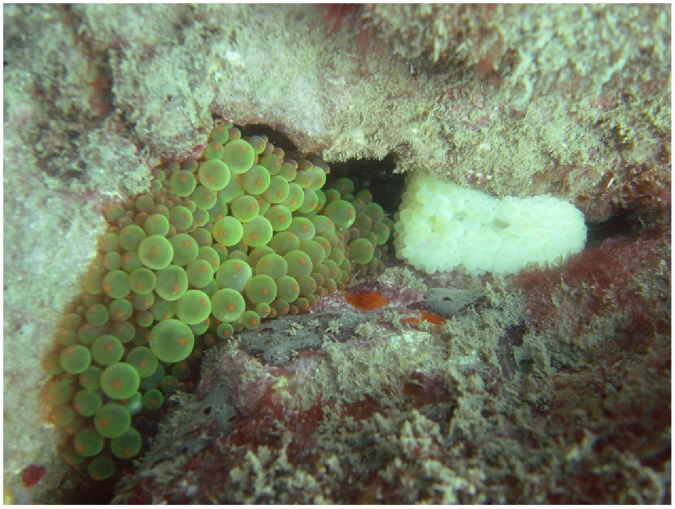
*Entacmaea quadricolor* anemones categorised as unbleached (left) and bleached (right) during the major bleaching event at the Keppel Islands, December 2011. The colour of the unbleached anemone is the typical colour of *E. quadricolor* at the Keppel Islands that is seen outside of major bleaching events.

Anemones were classified as bleached if more than 90% of the upper surface was pure white. We were not able to measure the loss of symbionts or photosynthetic pigments. However, in corals, colour intensity is linked to the density of *Symbiodinium* [30], and laboratory experiments have shown that anemone bleaching is characterised by a loss of symbionts and, in severe cases, a loss of photosynthetic pigments [27]. In coral bleaching field studies, the proportion of white on the upper tissue surface of the coral is a commonly used method to quantify spatial and taxonomic patterns of bleaching [31], [32]. The 90% threshold used in this study to classify anemones as bleached is equivalent to the upper range of the most severe bleaching category in coral bleaching studies [31], [32]. That is, only severely bleached anemones were categorised as bleached. In our observations, the majority of anemones can be classed as either severely bleached or unbleached (Figure 1). Unbleached anemones are those that have strong colouration typical of their descriptions in the literature [21] and are usually brown on their upper surface with tentacles that often have fluorescent or lightly-coloured tips. This study spanned a range of locations, observers and anemone species, and restricting the bleached category to only severely bleached anemones reduced subjectivity and observer bias.

### Sea Temperatures and Anemone Bleaching

To determine if anemone bleaching was associated with elevated sea temperatures we examined sea temperatures during major bleaching events (defined here as >10% of all anemones categorised as bleached). Coral bleaching events are classified based on the proportion of corals in a community that bleach: “moderate” events occur when 10 to 50% of corals bleach, “high” when 50 to 90% bleach, and “extreme” when more than 90% bleach [33], [34]. Here we use the term “major” to describe all the anemone bleaching events that fit into the “moderate”, ”high” and “extreme” categories used to describe coral bleaching events. There were five major bleaching events (Christmas Island 2005 and 2010, Keppel Islands 2011, Papua New Guinea 2009, and Thuwal 2010, Table 2) and we used published and unpublished data from *in situ* sea temperature data loggers and satellite derived sea surface temperature (SST) [35] to investigate the relationship between major bleaching events and elevated sea temperatures. To determine if the major anemone bleaching events coincided with coral bleaching events, we also assessed data (where available) on coral condition at the survey sites.

**Table 2:** The number and percentage of bleached host anemones at each study location.

Location	Species	% bleached	N
Marshall Islands, 2009	*E. quadricolor*	1.3	157
	*H. magnifica*	0	1
	*S. mertensii*	0	1
	Total	1.3	159
2010	*E. quadricolor*	1.8	334
	*H. aurora*	20	5
	*H. crispa*	0	3
	*H. magnifica*	0	1
	*S. mertensii*	0	4
	Total	2	347
Norfolk Island	*E. quadricolor*	0.1	2,269
	*S. haddoni*	0	2
	Total	0.1	2,271
Kosrae, 2010	*H. magnifica*	0	1
	*S. mertensii*	0	3
	Total	0	4
2012	*C. adhaesivum*	0	2
	*H. crispa*	0	4
	*H. magnifica*	0	4
	*S. haddoni*	0	1
	*S. mertensii*	0	8
	Total	0	19
Lord Howe Island, 2009	*E. quadricolor*	0.5	3,557
2011	*E. quadricolor*	0.2	2,138
	*H. crispa*	0	4
	Total	0.2	2,142
Middleton Reef	*E. quadricolor*	0	30
Elizabeth Reef	*E. quadricolor*	0	47
Pohnpei	*E. quadricolor*	0	126
	*H. magnifica*	0	1
	Total	0	127
North Solitary Island	*E. quadricolor*	0	278
	*H. crispa*	0	6
	Total	0	284
Mooloolaba	*E. quadricolor*	0	30
Keppel Islands, 2007	*E. quadricolor*	0.4	686
	*H. crispa*	0	6
	Total	0.4	692
**2011**	***E. quadricolor***	**19.4**	**1,762**
**Papua New Guinea**	***H. crispa***	**13.6**	**44**
	***S. haddoni***	**44.1**	**111**
	**Total**	**35.5**	**155**
Guam	*E. quadricolor*	0.7	137
	*H. crispa*	0	1
	Total	0.7	138
Ashmore Reef	*S. gigantea*	0	9
**Christmas Island, 2005**	***C. adhaesivum***	**9.1**	**22**
	***H. magnifica***	**30**	**20**
	**Total**	**19.5**	**42**
**2010**	***C. adhaesivum***	**70**	**40**
	***H. magnifica***	**2.6**	**38**
	***S. mertensii***	**33.3**	**3**
	**Total**	**37**	**81**
Cocos Islands, March 2008	*H. magnifica*	0	3
September 2008	*H. magnifica*	0	18
	Total	0	21
Farasan Banks, 2009	*E. quadricolor*	1.6	64
	*H. aurora*	0	5
	*H. crispa*	0	22
	*H. magnifica*	2.3	43
	*S. haddoni*	0	112
	Total	0.8	246
Farasan Banks, 2010	*E. quadricolor*	7.1	14
	*H. aurora*	0	2
	*H. crispa*	0	5
	*H. magnifica*	0	14
	*S. haddoni*	0	56
	Total	1.1	91
Thuwal-Yanbu	*E. quadricolor*	0	11
	*H. aurora*	0	2
	*H. crispa*	0	2
	*H. magnifica*	0	45
	*S. haddoni*	0	89
	Total	0	149
**Thuwal**	***E. quadricolor***	**100**	**6**
	***H. magnifica***	**100**	**6**
	***S. haddoni***	**100**	**3**
	**Total**	**100**	**15**
Total all locations	All species	3.5	13,896

Anemone data is combined for all sites and depths (3 to 20 m) at each location. The five major anemone bleaching events are indicated in bold.

### Spatial Bleaching Patterns

To allow for effective statistical comparisons, analyses focused on determining patterns of bleaching during the major bleaching events (listed above). To determine spatial patterns in bleaching, the numbers of bleached and unbleached host anemones were compared between sites using chi-square tests. All anemones bleached during the Thuwal 2010 event so data from this location were not included in this analysis. Chi-square tests were also used to compare the number of bleached and unbleached host anemones at different depths. Where possible, each host anemone species was analysed separately; however, if the data violated the assumptions of the analysis (more than 20% of cells with expected counts <5), then data were combined for all species. Yates’ correction for continuity was applied for 2×2 contingency tables. During the major bleaching event in Papua New Guinea in 2009, the depth of each anemone was recorded (rather than transects at specific depths), so *t*-tests were used to compare the mean depth of bleached versus unbleached anemones for each species. Statistical analyses were performed using SPSS (version 17, Chicago U.S.A) or R [36].

### Bleaching Consequences

A variety of approaches were used to determine whether the abundance or size of host anemones decreased during and following a major bleaching event. For Christmas Island (2005 and 2010 bleaching events), the substrate adjacent to bleached anemones was tagged (numbered cattle ear tags) [37] or the exact locations of anemones were mapped [28], [29]. Follow-up surveys (2–4 months after bleaching) determined if bleached anemones subsequently disappeared. For the bleaching event in Papua New Guinea (2009), bleached and unbleached anemones (*S. haddoni*) were tagged and photographed before and during the bleaching event and changes in size (surface area) were measured from the photographs (see [38] for detailed methodology).

For the major bleaching event in the Keppel Islands we monitored *E. quadricolor* colonies. This anemone species has a solitary and colonial morph [39]; however, only the colonial morph was observed at the Keppel Islands [40]. Within colonies, individual anemones were no more than 30 cm from each other. The number of *E. quadricolor* individuals in a colony (marked by GPS), was recorded before (2007 or 2009), during (2011) and after the bleaching event (2012). The same colonies were monitored through time, but the number of colonies that were monitored increased with time. Five colonies were measured in 2007, an additional three in 2009, and 10 additional colonies were surveyed from 2011 onwards. To increase sample size for analyses, data from 2007 and 2009 were combined into the “before bleaching” survey period.

During the Keppel Island bleaching event, individual *E. quadricolor* anemones either had normal colouration (dark brown with tentacles often exhibiting fluorescent green and orange tips) or had completely bleached white (i.e. not pale brown or partly white) (Figure 1). Colonies were categorised as unbleached if all individual anemones in the colony had normal colouration, and bleached if all individuals in the colony were white. A small proportion (9%) of colonies contained bleached and unbleached anemones and these were not included in the analyses, except for one colony where approximately 80% of individuals were bleached. This colony was included in the bleached group, but only the sizes of bleached individuals were recorded.

Anemone diameter (tentacle crown long diameter [22], [37]) of 10 individuals per colony was measured *in situ* using a tape-measure to provide an estimate of the average diameter of individuals in each colony. We did not use the photographic approach of measuring surface area (as above for *S. haddoni* [38]) because it was difficult to accurately measure *E. quadricolor* anemones from photographs. This is because the surface area is often obscured as individuals are clumped into colonies and are often among dead branching corals. Accurate measurements of anemones are difficult because they have no hard structures and an individual is able to expand and contract in size (including tentacle crown diameter). However, using the average diameter of 10 individuals per colony and measuring numerous colonies across multiple sites and survey periods provides a broad and approximate test of temporal changes in colony size related to bleaching status that are less likely to be affected by changes in body size of an individual that may occur at any time.

To test if the abundance and size of anemones in the Keppel Islands declined through time due to bleaching, we constructed a suite of linear mixed-effects models using the nlme library [41] in R [36] for each of the response variables - abundance and size of anemones. We included two explanatory variables, “bleaching category” (bleached or unbleached) and “time”, as individual effects, as combined effects, and as an interaction between effects. As anemone colonies were sampled repeatedly over time, individual colonies were coded as a random effect to account for the temporal dependence structure between the observations. We compared and ranked models using weights of Akaike’s information criterion corrected for small sample size (*w*AIC*_c_*). AIC*_c_* weight varies from 0 (no support) to 1 (complete support) relative to all models in the set [42].

## Results

### Sea Temperature and Bleaching

**Figure 2:**
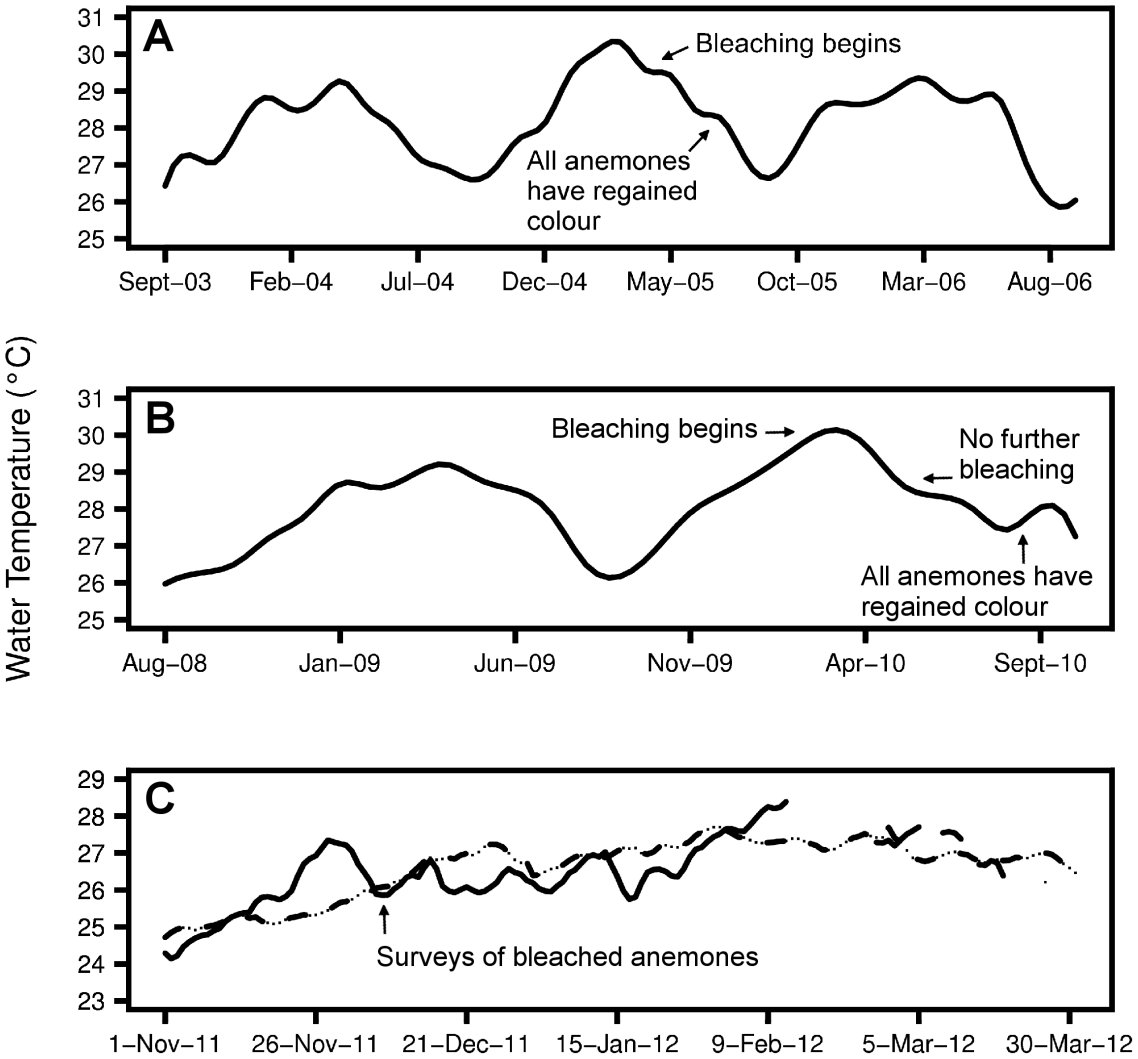
Seawater temperature during anemone bleaching events. (A) Archival sea surface temperature for Christmas Island obtained from Daily Optimal Interpolation dataset ([34] available at http://coastwatch.pfeg.noaa.gov). *in situ* measurements of water temperature at (B) 11 m depth at Christmas Island and (C) at 6 m depth at Keppel Islands, based on Australian Institute of Marine Science data. These temperature datasets coincide with major bleaching events at (A) Christmas Island 2005, (B) Christmas Island 2010 and (C) Keppel Islands 2011. For the Keppel Islands (C), the dotted-dashed line represents the summer average (2007–2011), while the solid line represents the 2011–2012 summer. The survey period (December 2011) is indicated with an arrow. Temperature data were unavailable for short periods in February and March 2012.

**Figure 3:**
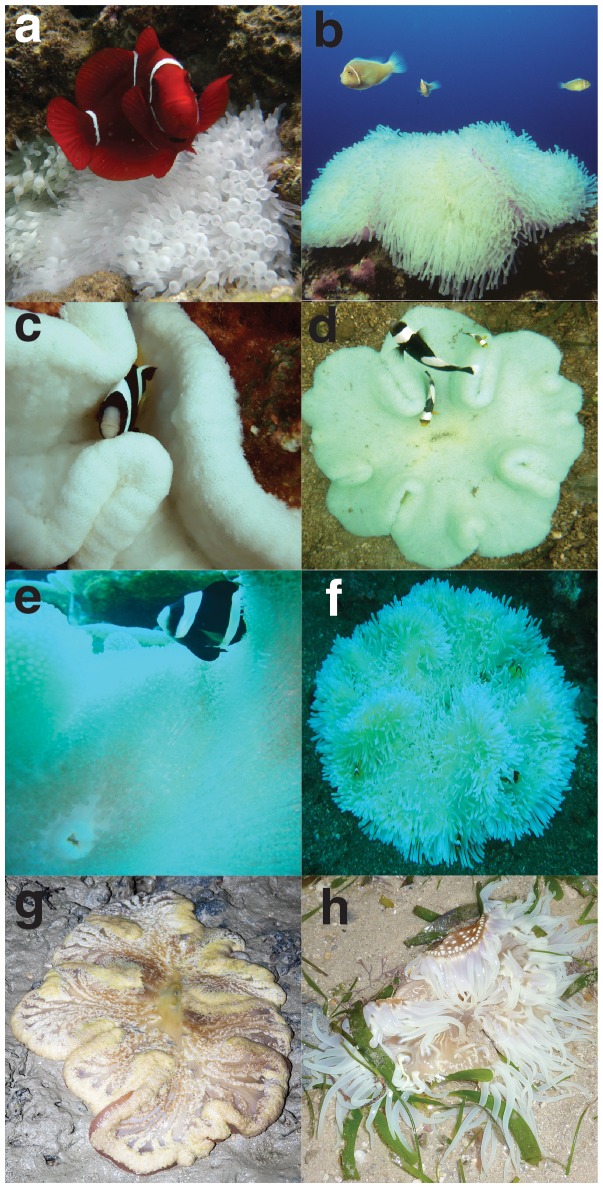
Bleached host anemones. Bleached anemones with resident anemonefish: (A) *Entacmaea quadricolor* inhabited by *Premnas biaculeatus* (Papua New Guinea) (B) *Heteractis magnifica* inhabited by *Amphiprion perideraion* (Christmas Island); (C) *Cryptodendrum adhaesivum* inhabited by *A. clarkii* (Christmas Island); (D) *Stichodactyla haddoni* inhabited by *A. polymnus* (Papua New Guinea); (E) *S. mertensii* inhabited by *A. clarkii* (Christmas Island); (F) *H. crispa* inhabited by *A. clarkii* (Bali); (G) *S. gigantea* (Singapore); (H) *Macrodactyla doreensis* (Singapore). Photograph credits: P. Saenz-Agudelo (A, D), J. Gilligan (B), J-P Hobbs (C,E), A. Scott (F), R. Tan (G), K. Sheng (H).

Archival SST data and *in situ* water measurements confirmed that all five of the major anemone bleaching events were associated with elevated water temperatures (Figure 2, [38], [43]). Furthermore, bleached corals were observed during four of the five anemone bleaching events. Bleached corals were not seen during the fifth event (Keppel Islands, 2011), but coral cover was unusually low due to past bleaching events. In addition to bleaching events that we witnessed, there was widespread bleaching of host anemones in Singapore in June 2010 (Figure 3G,H) during a thermally-induced coral bleaching event (Kok Sheng, pers. comm., [44]).

### Taxonomic Bleaching Patterns

Of the 13,896 anemones (all host species) that were observed at all survey locations, 490 (3.5%) were bleached (Table 2). Of the eight species of host anemone seen during surveys, seven were found to bleach: *Cryptodendrum adhaesivum*, *Entacmaea quadricolor*, *Heteractis aurora, H. crispa*, *H. magnifica, Stichodactyla haddoni* and *S. mertensii* (Figure 3). The eighth species, *S. gigantea*, was rarely seen during our surveys (*n* = 9 individuals), and although none were bleached, this anemone has been observed to bleach elsewhere (Figure 3G). The remaining two host anemones, *Macrodactyla doreensis* and *H. malu*, were not observed during this study. However, bleached *M. doreensis* have been observed in Singapore (Figure 3H) and bleached *H. malu* have been observed in aquaria (J-P Hobbs, pers. obs.).

Species-specific susceptibility of host anemones during the five major bleaching events was variable and dependent upon location and year. For example, in Papua New Guinea (2009), 13.6% of *H. crispa* bleached compared to 44.1% of *S. haddoni* (Table 2). During the bleaching event at Thuwal (2010), three species of host anemones were observed (*E. quadricolor*, *H. magnifica,* and *S. haddoni*) and all individuals were bleached (Table 2). During the 2005 bleaching event at Christmas Island, 30% of *H. magnifica* bleached but only 9.1% *C. adhaesivum* bleached. However, the opposite pattern of susceptibility was seen at the same location in 2010 where 70% of *C. adhaesivum* bleached compared to 2.6% of *H. magnifica* (Table 2).

### Spatial Bleaching Patterns

Bleaching of host anemones was observed at 10 of the 19 locations and was geographically widespread, from Saudi Arabia in the west to the Marshall Islands in the east (Table 2). Bleaching extended latitudinally from near the equator (Singapore and Papua New Guinea) to the world’s southernmost coral reefs (Lord Howe Island, 32°S). Bleached anemones were recorded at both oceanic and continental locations.

**Figure 4:**
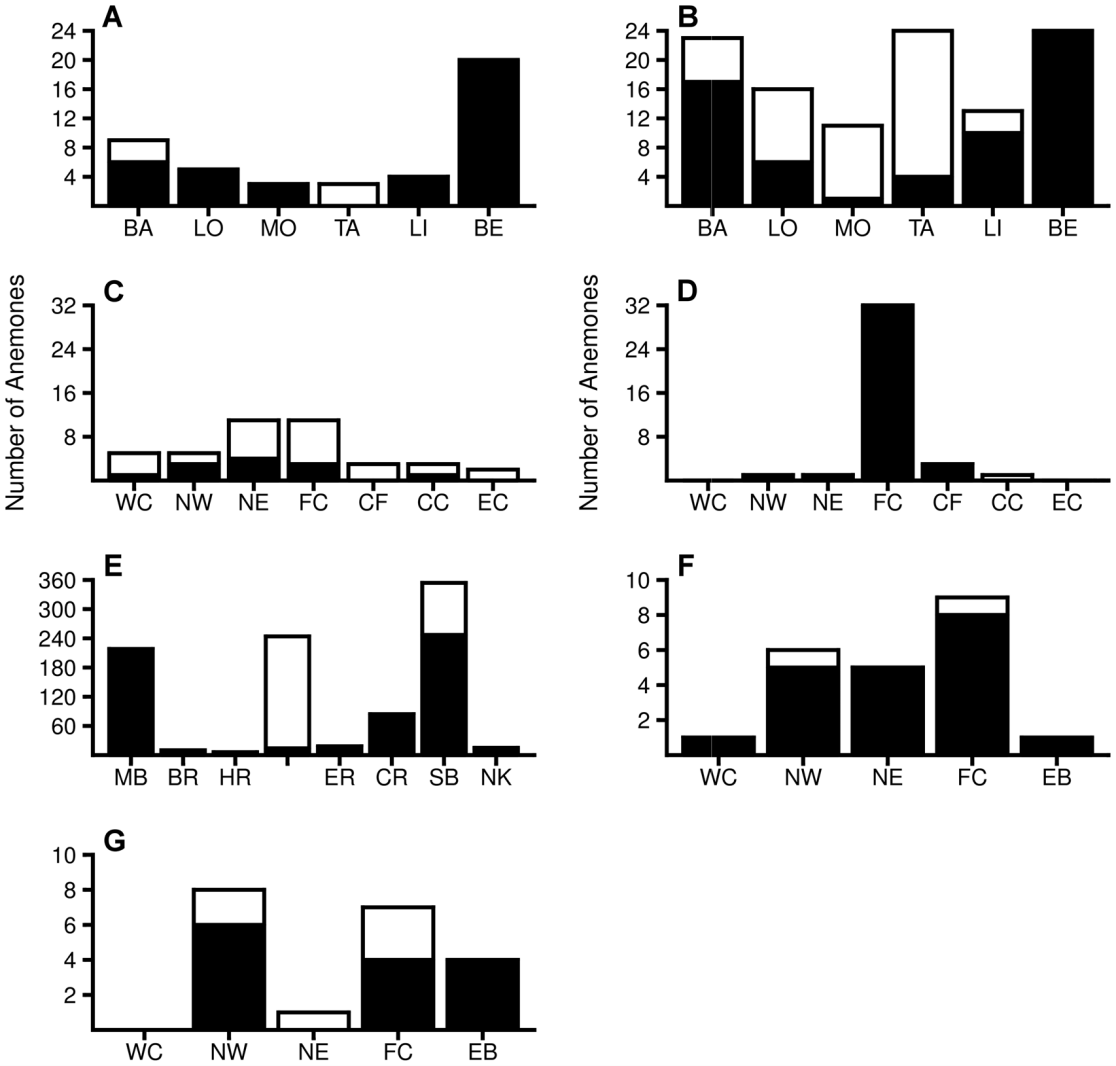
Spatial variation in anemone bleaching. Number of bleached (white bars) and unbleached (black bars) host anemones encountered in surveys at different sites during the major anemone bleaching events. Data are presented for: (A) *Heteractis crispa* and (B) *Stichodactyla haddoni* at Papua New Guinea in 2009; (C) *Cryptodendrum adhaesivum* and (D) *H. magnifica* at Christmas Island, 2010; (E) *Entacmaea quadricolor* at Keppel Islands, 2011; (F) *C. adhaesivum* and (G) *H. magnifica* at Christmas Island, 2005. Abbreviations for site names are provided in Table S1.

In three of the four major bleaching events, there was considerable variation in the proportion of bleached anemones among sites. During the 2009 bleaching event in Papua New Guinea, bleaching in *H. crispa* and *S. haddoni* differed substantially among sites, affecting 0 to 85% of anemones depending on the site (both species combined: χ^2^ = 65.706, df = 5, p<0.0001, Figure 4A, B). At Christmas Island in 2010, the proportion of bleached *C. adhaesivum* and *H. magnifica* varied considerably (19 to 100%) among sites (both species combined: χ^ 2^ = 18.866, df = 6, p = 0.004, Figure 4C, D). At the Keppel Islands in 2011, bleaching in *E. quadricolor* varied from 0 to 94% among sites (χ^ 2^ = 556.439, df = 7, p<0.0001, Figure 4E). However, at Christmas Island in 2005 the proportion of bleached *C. adhaesivum* (0–13%) and *H. magnifica* (0–75%) did not differ significantly among sites (both species combined: χ^ 2^ = 1.853, df = 4, p = 0.763, Figure 4F, G). It was not possible to test for spatial patterns in the fifth major bleaching event (Thuwal, 2010) because all surveyed anemones were bleached.

**Figure 5:**
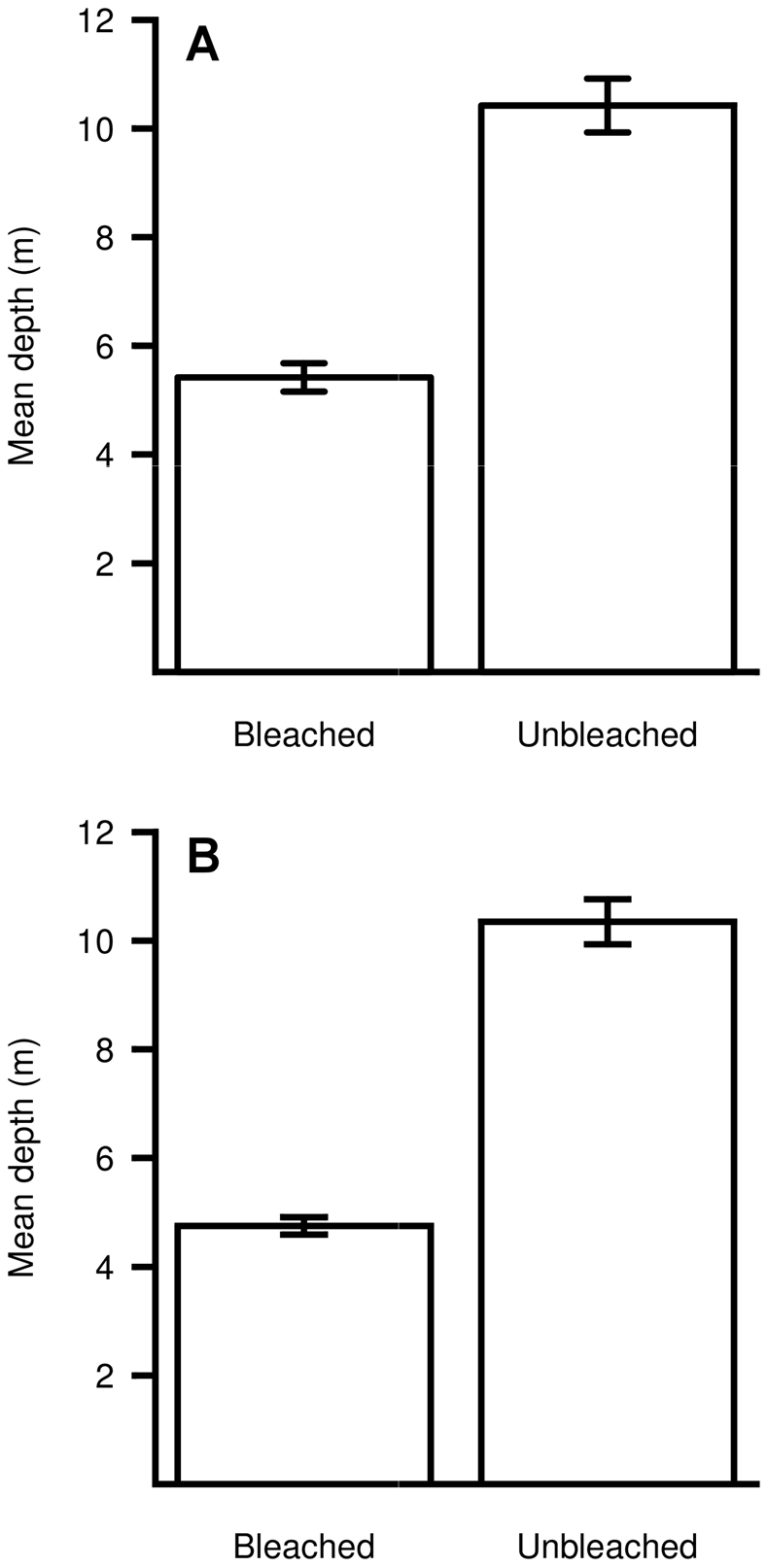
Depth variation in anemone bleaching at Papua New Guinea. Mean depth (±SE) of bleached and unbleached host anemones during the major bleaching event at Papua New Guinea in 2009. (A) *Heteractis crispa* and (B) *Stichodactyla haddoni*.

**Figure 6:**
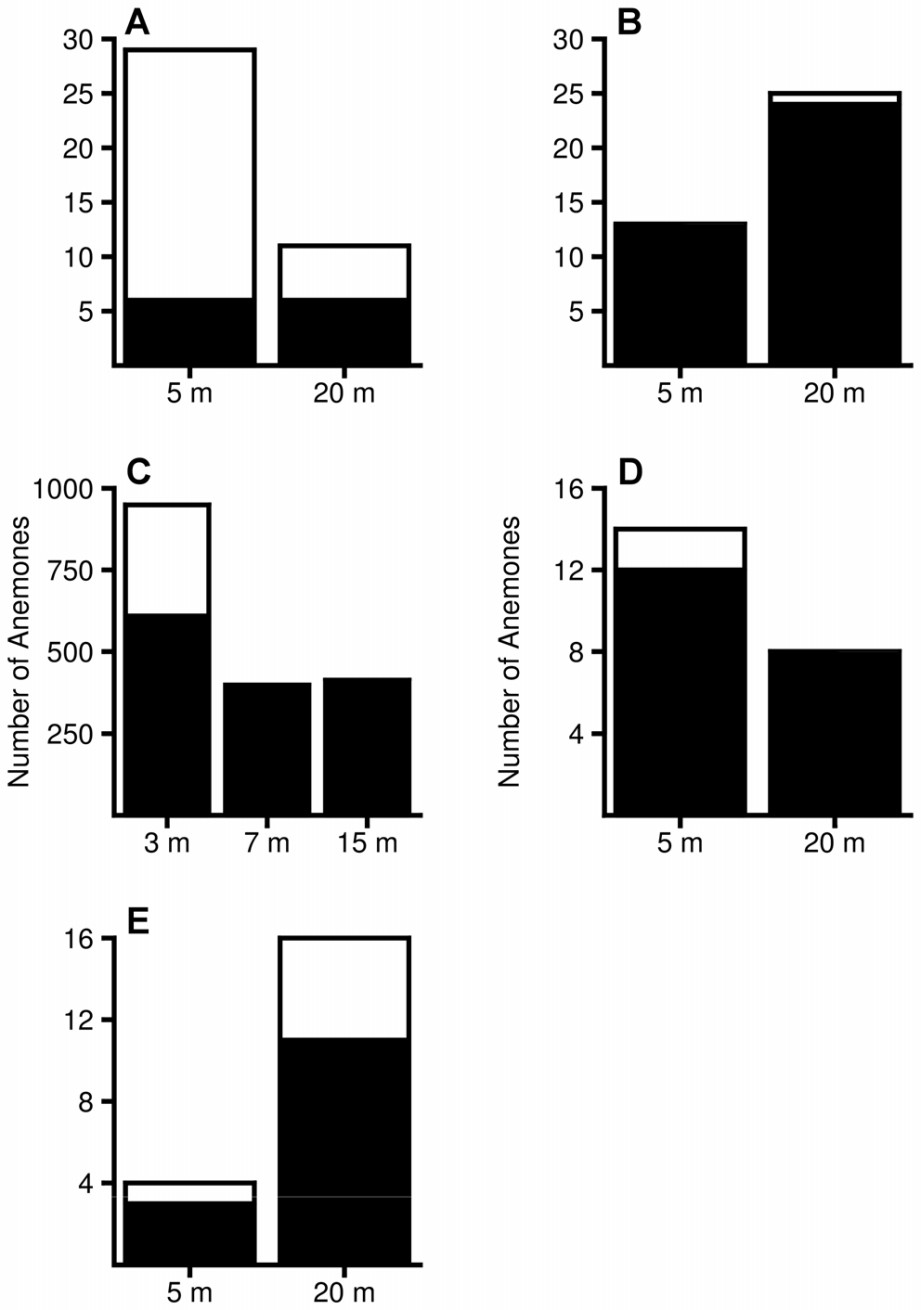
Depth variation in anemone bleaching at Christmas Island and the Keppel Islands. Number of bleached (white bars) and unbleached (black bars) host anemones encountered in surveys at different depths during the major anemone bleaching events. Data presented for: (A) *Cryptodendrum adhaesivum* and (B) *Heteractis magnifica* at Christmas Island, 2010; (C) *Entacmaea quadricolor* at Keppel Islands, 2011; (D) *C. adhaesivum* and (E) *H. magnifica* at Christmas Island, 2005.

Anemone bleaching decreased with depth in most of the major bleaching events. At Papua New Guinea (2009) the mean depth of bleached *H. crispa* (5.4 m ±0.3 SE) was significantly shallower than that of unbleached *H. crispa* (10.4 m ±0.5 SE) (*t*-test: *t* = 8.9 df = 38, p<0.0001, Figure 5A). Similarly, the mean depth of bleached *S. haddoni* (4.8 m ±0.2 SE) during this event was shallower than that for unbleached *S. haddoni* (10.3 m ±0.4 SE) (*t*-test: *t* = 12.6, df = 78, p<0.0001, Figure 5B). At Christmas Island in 2010, bleaching in *C. adhaesivum* and *H. magnifica* was three times greater in the shallows (both species combined: 54% bleached at 5 m compared to 17% at 20 m, Yates’ correction χ^ 2^ = 10.469, df = 1, p = 0.0012, Figure 6A, B). At the Keppel Islands in 2011, 36% of *E. quadricolor* were bleached at 3 m depth, compared to less than 1% at 7 and 15 m depth (χ^ 2^ = 354.396, df = 2, p<0.0001, Figure 6C). At Christmas Island in 2005, the proportion of *C. adhaesivum* and *H. magnifica* that bleached was not significantly different between the two depths examined (both species combined: Yates’ correction χ^ 2^ = 0.003, df = 1, p = 0.956, Figure 6D, E).

### Bleaching Consequences

**Figure 7:**
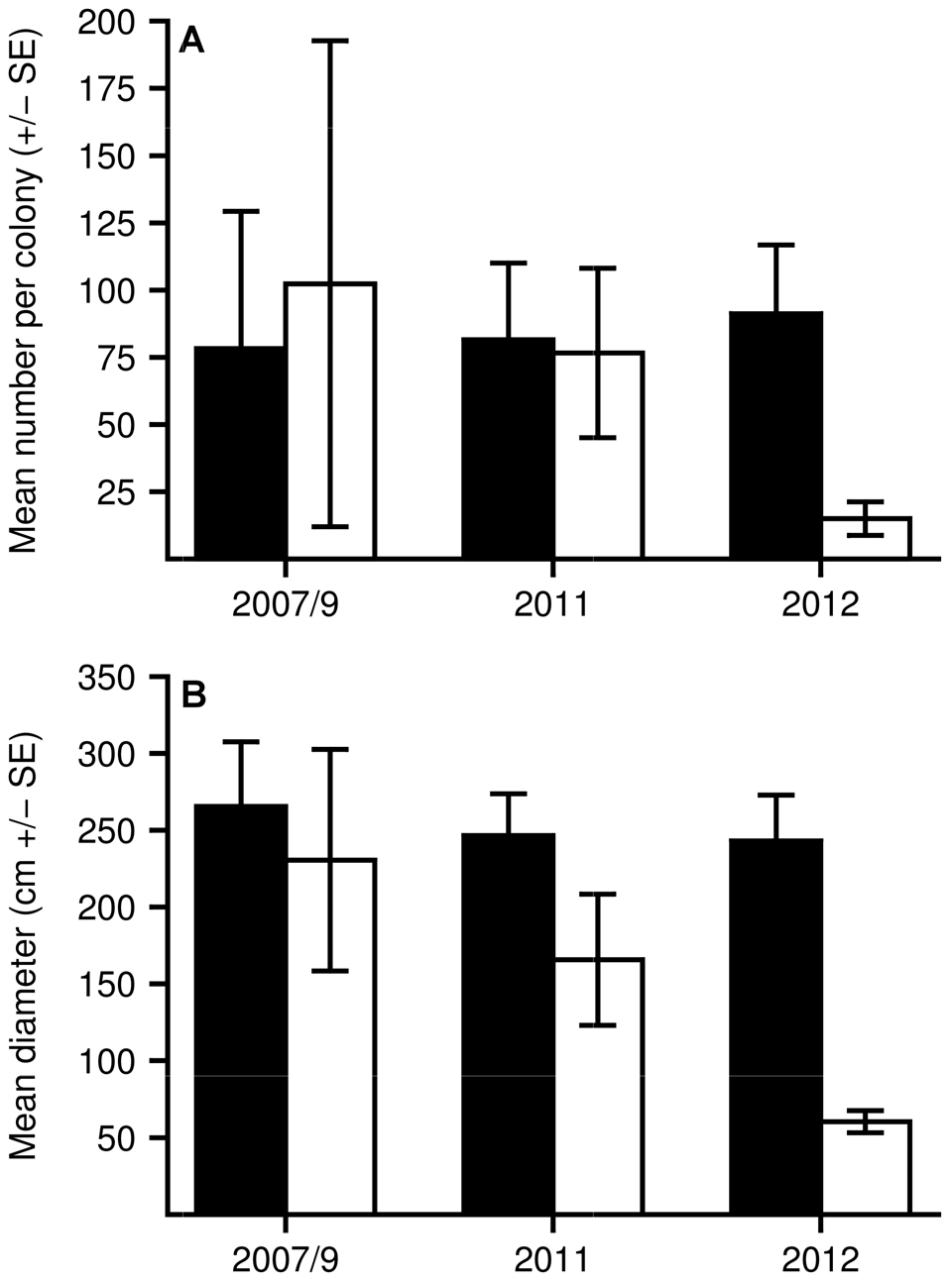
Changes in the abundance and size of anemones following a bleaching event. (A) Mean number (±SE) of anemones per colony, and (B) the mean diameter (±SE) of individual anemones within each colony for *Entacmaea quadricolor* before (2007, 2009), during (2011), and after (2012) the major anemone bleaching event at the Keppel Islands. Colonies that bleached during the 2011 event are represented by white bars and colonies that did not bleach are represented by black bars. To increase sample size for analyses, data for 2007 and 2009 were combined into the before bleaching period.

Reductions in the abundance and size of host anemones were detected following bleaching events. After the bleaching event in Papua New Guinea (2009), the size of bleached *S. haddoni* decreased on average by 34% and 1% (3/256) of anemones disappeared. At the Keppel Islands (2011), comparisons between bleached and unbleached *E. quadricolor* anemones revealed that for both abundance and size, the model including the interaction between bleaching category and time had 100% support (*w*AIC*_c_* = 1.0) (Table 3 and 4). That is, following the bleaching event, bleached *E. quadricolor* anemones declined noticeably in abundance (85% decline) and size (74% decline) (Figure 7A,B). Abundance and size remained relatively unchanged for unbleached anemones.

**Table 3:** Ranked linear mixed-effects models of anemone abundance explained by date, bleaching category (bleach_cat) and the interaction between these two variables.

mod	*k*	LL	AIC*_c_*	ΔAIC*_c_*	*w*AIC*_c_*
Abundance∼date×bleach_cat+(1|id)	6	−199.7	417.79	0	1
Abundance∼date+bleach_cat+(1|id)	4	−212.6	438.24	20.45	<0.01
Abundance∼date+(1|id)	3	−217.5	445.6	27.81	<0.01
Abundance∼bleach_cat+(1|id)	2	−221.9	452.19	34.4	<0.01
Abundance∼1+(1|id)	1	−226.9	459.82	42.03	<0.01

All models have individual colony coded as a random effect (1|id). The models are ranked against the intercept-only model (NULL model). Also shown is the number of estimable model parameters (*k*), maximum log-likelihood (LL), Akaike’s information criterion corrected for small samples (AIC*_c_*), the difference in AIC*_c_* for each model from the top-ranked model (ΔAIC) and the model weight (*w*AIC*_c_*).

**Table 4:** Ranked linear mixed-effects models of anemone size explained by date, bleaching category (bleach_cat) and the interaction between these two variables.

Model	*k*	LL	AIC*_c_*	ΔAIC*_c_*	*w*AIC*_c_*
Size∼date×bleach_cat+(1|id)	6	−186	390.73	0	1
Size∼date+bleach_cat+(1|id)	4	−200.5	414.31	23.57	<0.01
Size∼date+(1|id)	3	−208.8	428.28	37.55	<0.01
Size∼bleach_cat+(1|id)	2	−211.9	432.15	41.42	<0.01
Size∼1+(1|id)	1	−219.7	445.57	54.84	<0.01

All models have individual colony coded as a random effect (1|id). The models are ranked against the intercept-only model (NULL model). Also shown is the number of estimable model parameters (*k*), maximum log-likelihood (LL), Akaike’s information criterion corrected for small samples (AIC*_c_*), the difference in AIC*_c_* for each model from the top-ranked model (ΔAIC) and the model weight (*w*AIC*_c_*).

At Christmas Island (2005 and 2010) there was no change in abundance of bleached anemones (anemone size was not measured). In 2005, three bleached *C. adhaesivum* and seven bleached *H. magnifica* were monitored and all regained normal colour within two months, and in 2010, 10 bleached *C. adhaesivum*, two bleached *H. magnifica* and one bleached *S. mertensii* all regained colour within four months.

## Discussion

This study confirms that anemone bleaching events are associated with elevated sea temperatures. Nine of the ten species of host anemones bleached in their natural environment, while bleaching in the tenth species was observed in aquaria. However, as in corals, the effects of anemone bleaching are not uniform because bleaching susceptibility varies among species and bleaching events. Bleaching can occur throughout the geographic ranges of host anemones, including inshore and offshore locations. During most of the bleaching events, bleaching varied among sites and was greater in the shallows, indicating that some small-scale spatial refuges exist. Bleaching also led to a decrease in size and abundance of some host anemone species.

Although surveys were conducted at various seasons, years, and locations, there was a clear pattern that anemone bleaching was linked with increased sea temperatures. The role of elevated water temperature in causing bleaching in host anemones has been confirmed by laboratory studies [27]. Furthermore, major anemone bleaching events coincide with thermally-induced coral bleaching events [28], [29](this study). Thus, anemone bleaching and coral bleaching appear to be caused by the same environmental factors [27]. However, a small proportion (<0.3% or 40/13896) of bleached anemones were observed at times when sea temperatures were not elevated. This suggests that anemone bleaching can also occur in response to stressors other than elevated water temperature. For example, decreased salinity can cause bleaching in corals [45], and probably explains the bleaching and mortality of host anemones observed during the 2007 flood event in Singapore (Kok Sheng, pers. comm.).

Some species of host anemone were more prone to bleaching than others. In corals, species-specific bleaching susceptibility has been attributed to differences in: physical characteristics of the colony [6], [46]; thermal sensitivity of symbionts [47], [48]; diversity of symbionts [49], prior bleaching history [44] and host-symbiont relationship [50]. Whether these factors influence taxonomic bleaching patterns in host anemones is unknown. There was some indication that prior bleaching history may influence anemone bleaching at Christmas Island where taxonomic patterns of susceptibility changed between bleaching events (i.e. previously bleached species were less likely to bleach a second time). Despite differences in taxonomic susceptibility, the Thuwal 2010 bleaching event illustrated that elevated sea temperatures can exceed thermal tolerances and cause bleaching in all individuals of all host anemones species. This indicates that taxonomic differences in bleaching susceptibility are evident during moderate bleaching events, but not during severe events.

Bleaching can occur throughout the geographic range of host anemones, and can extend from the equator to the latitudinal limits of coral reefs [28], [29] (this study). However, there was considerable local-scale spatial variation in bleaching. In corals, spatial differences in bleaching may be due to variation in a range of intrinsic and extrinsic factors and interactions between factors [6], [47], [50]–[53]. In this study, we did not notice any obvious differences between sites that might explain spatial differences in anemone bleaching. Regardless of the causative agent(s), the heterogeneous nature of anemone bleaching suggests that some sites serve as refuges during bleaching events.

Bleaching of host anemones decreased with depth during most of the major bleaching events, indicating that bleaching is depth-dependent. Coral bleaching is thought to be more common in the shallows (see review by [12]), with deeper reefs appearing to be a refuge for some species (e.g. [11], [54]). This is likely to be due to depth-related gradients in temperature and solar irradiance [14], [15]. Elevated temperatures and high irradiance cause bleaching for at least one species of host anemone [27] and reductions in both of these factors with depth may explain why anemone bleaching decreases with depth. Host anemones have also been reported on mesophotic reefs below 50 m depth [55] and this habitat may be a critical refuge in a future of rising sea temperatures.

The effects of bleaching on anemone abundance and size differed between species and bleaching events. The abundance of *E. quadricolor* decreased following the bleaching event at the Keppel Islands (and in Japan: [29]), but there was no decrease in abundance of bleached *C. adhaesivum*, *H. magnifica* and *S. mertensii* at Christmas Island. There was little change in abundance of *H. crispa* following the bleaching event in Papua New Guinea (2009), but this species decreased considerably in abundance after a bleaching event in Japan [28]. These mixed results may be due to differences in the thermal characteristics of the bleaching event (e.g. magnitude and duration of elevated water temperatures), prior bleaching history, or geographical differences in some other intrinsic and extrinsic factors (see above). Further studies are required to determine if and how these factors influence species-specific patterns of bleaching susceptibility.

Decreases in anemone abundance immediately following bleaching events probably indicate increased mortality. Although anemones occasionally move or retreat into the reef matrix, we are confident that missing anemones died rather than moved or retreated because we (1) marked the location of anemones (with a tag or GPS), (2) periodically monitored their condition and location, and (3) thoroughly searched the reef matrix if an anemone could not be found. Little or no movement of anemones has been recorded in other studies using the same methods [28], [29], [37]. In addition, we observed bleached *E. quadricolor* at the Keppel Islands and Lord Howe Island remain in the same location and slowly shrink over several weeks and then die (turn into amorphous white slime). In each case, resident anemonefish remained in the vicinity of retreated anemones, but they too disappeared when the anemone died.

Increased mortality (disappearance) rates in host anemones have followed bleaching events elsewhere. For example, in Okinawa the mortality (disappearance) rate of *H. crispa* before a bleaching event was between 4 and 5% per year, and in the year after the bleaching event the rate was 73% [28]. At the same location, *E. quadricolor* had the same abundance for a 6-year pre-bleaching period, but abundance decreased substantially (25% in a year) after the bleaching event [29]. Host anemones tend to be long lived (decades to more than 100 years old), with low mortality rates [21] and thus are particularly vulnerable to increases in mortality rates. Furthermore, host anemones appear to have low recruitment rates. Anemone recruitment at Okinawa was approximately 4% and 2% per year (based on original population size) for *H. crispa* and *E. quadricolor*, respectively [28], [29]. As long-lived species with low natural mortality and recruitment rates, bleaching events could pose a serious threat to population viability of host anemones.

Decreases in the size of host anemones following bleaching events may also compromise population viability. Bleaching in corals can limit reproduction [2], [56] and reduce colony size, which may further decrease reproductive output [57]–[59]. Although reductions in size were observed in some anemone species following bleaching, the effect on reproduction is unknown. Any detrimental consequences to anemone reproduction would limit the ability for populations to recover following bleaching events. There may also be other unknown negative effects of bleaching (e.g. reduced growth and immunity have been reported for bleached corals: [20], [60]–[62]) that may further threaten the viability of host anemone populations.

Anemonefishes have an obligate relationship with host anemones and decreases in the abundance and size of anemones can lead to population declines or local extinction of anemonefishes [28], [29]. Decreases in both the number of adult pairs and the size of individuals forming adult pairs can occur following anemone bleaching events [28], [29], which is likely to reduce reproductive output of anemonefishes. In addition, decreases in the clutch size of anemonefishes can occur during anemone bleaching events [38]. Collectively, this confirms that anemone bleaching can have negative cascading effects on anemone ectosymbionts.

This study illustrates that host anemone species are differentially susceptible to bleaching and that anemone bleaching is geographically widespread. The occurrence of major anemone bleaching events in response to episodes of elevated sea temperatures is concerning given the forecast increase in sea temperatures. Thus, if host anemones (and their symbiotic zooxanthellae) cannot acclimate or adapt to rising sea temperatures, then populations of host anemones and associated anemonefishes are anticipated to decline significantly. Although symbiosis contributes to the iconic status and commercial importance of anemones and anemonefishes, it may also prove to be their downfall.

## Supporting Information

Table S1 Names of survey sites and the corresponding abbreviations used in Figure 2.
(DOCX)
