# Supplementary material for: Taxonomic, Spatial and Temporal Patterns of Bleaching in Anemones Inhabited by Anemonefishes
Source: PLoS One. 2013 Aug 8;8(8):e70966. doi: 10.1371/journal.pone.0070966 (PMC3738586; doi:10.1371/journal.pone.0070966)
Supplement: Table S1 — Names of survey sites and the corresponding abbreviations used in Figure 2. (DOCX) [file pone.0070966.s001.docx]

**Table S1:** Names of survey sites and the corresponding abbreviations used in Figure 2.

| **Location (year)** | **Survey site name** | **Abbreviation** |
| --- | --- | --- |
| Papua New Guinea (2009) | Loloata South Bank | BA |
| Papua New Guinea (2009) | Loloata Jetty | LO |
| Papua New Guinea (2009) | Motupore Island | MO |
| Papua New Guinea (2009) | Taurama | TA |
| Papua New Guinea (2009) | Lion Island | LI |
| Papua New Guinea (2009) | Manubada Island | BE |
| Christmas Island (2010) | West Coast | WC |
| Christmas Island (2010) | North-West Coast | NW |
| Christmas Island (2010) | North-East Coast | NE |
| Christmas Island (2010) | Flyingfish Cove | FF |
| Christmas Island (2010) | Chicken Farm | CF |
| Christmas Island (2010) | Casino Coast | CC |
| Christmas Island (2010) | East Coast | EC |
| Keppel Islands (2011) | Monkey Beach | MB |
| Keppel Islands (2011) | Bald Rock | BR |
| Keppel Islands (2011) | Half-tide Rock | HR |
| Keppel Islands (2011) | Middle Island | MI |
| Keppel Islands (2011) | Egg Rock | ER |
| Keppel Islands (2011) | Conical Rocks | CR |
| Keppel Islands (2011) | Shelving Beach | SB |
| Keppel Islands (2011) | North Keppel Island | NK |
| Christmas Island (2005) | West Coast | WC |
| Christmas Island (2005) | North-West Coast | NW |
| Christmas Island (2005) | North-East Coast | NE |
| Christmas Island (2005) | Flyingfish Cove | FC |
| Christmas Island (2005) | Ethel Beach | EB |
